# *In Vitro* Antibacterial Activity of Different Honey Samples against Clinical Isolates

**DOI:** 10.1155/2022/1560050

**Published:** 2022-01-21

**Authors:** Mahasin Ahmed Wadi

**Affiliations:** Medical Microbiology, Department of Medical Surgical College of Nursing, Princess Nourah Bint Abdulrahman University, Riyadh, Saudi Arabia

## Abstract

**Background:**

The emergence of multi-drug-resistant organisms has created a lot of clinical problems. Hence, there is a need to find natural alternative treatment to counter the multi-drug-resistant organisms. Honey has a well-established usage as wound dressing in ancient and traditional medicine.

**Objective:**

The objective of this study is to establish a baseline for the antibacterial activity of 32 global raw natural and commercial various honey samples against 8 clinical isolates.

**Methods:**

Thirty-two honey samples (raw and commercial honey) collected from different global countries with different floral origins were tested *in vitro* for antibacterial activity against 8 clinical isolates collected from patients, at private hospital from Sudan, using disk diffusion technique. The following 6 epsilometer tests (Etest), amoxicillin, ciprofloxacin, cefotaxime, chloramphenicol, gentamicin, and tetracycline, were used against 8 clinical isolates for Minimum Inhibitory Concentration (MIC).

**Results:**

The following 8 clinical isolates were identified by conventional bacteriological methods: *Staphylococcus aureus, (S. aureus) Escherichia coli* (*E. coli*), *Klebsiella pneumoniae* (*K*. *pneumoniae*), *Pseudomonas aeruginosa* (*P*. *aeruginosa*), *Proteus vulgaris* (*P*. *vulgaris*), *Salmonella* Typhi (*S.* Typhi), *Shigella sonnei* (*S*. *sonnei*), and methicillin-resistant *Staphylococcus aureus* (MRSA). Both raw natural and commercial honey exhibited antibacterial properties against tested Gram-positive and Gram-negative bacteria. The tested organisms showed low sensitivity to antibiotic Etest.

**Conclusion:**

All of the bacterial species studied were uniformly receptive to all raw and commercial tested honey samples; in contrast, the tested organisms showed low sensitivity to antibiotics. Commercial honey has the same antibacterial activity as the raw natural unprocessed honey against tested clinical isolates. Thus, honey is a successful alternative to conventional antibiotics as has been proved against clinical isolates.

## 1. Introduction

Honey is a viscous sugar solution made by honey bees from plant nectar and honeydew. Honey has been used for medicinal purposes for centuries and has been reintroduced in modern medical practice. Composition of honey differs depending on the plant source, but the key components of the bee forage are identical to those found in all honeys [1].

Honey antimicrobial activity is the main factor for wound protection due to osmolarity effect, elevated concentration of sugar, low water content, and low pH [2, 3].

Topical application of honey to the first- and second-degree burns was found to be effective in decreasing morbidity and reducing time duration required for recovery [4].

Australian Jelly bush honey is a commercial honey which has been marked as bioactive honey. Antibacterial activity was due to other causes, such as bee-defensin-1 peptides and phenolic compounds [5].

Honey exerted bacteriostatic and bactericidal activity against methicillin-resistant *S*. *aureus* isolates from infected wound [6].

Some honeys demonstrate a wide range of antimicrobial activities against resistant bacteria [7].

Phenyllactic acid and methyl syringate were considered as fingerprint and chemical markers of Agastache honey and honey of Leptospermum sources [8]. Free phenols (volatile compounds), phenolic acids, polyphenols (usually in the form of flavonoids), anthocyanins, procyanidins, and pigments are found in the nectar honey phenolic compounds [9]. Manuka honey used in wound-care products may tolerate dilution with large amounts of wound exudate while still remaining active enough to suppress bacterial development [10].

Broad-spectrum antimicrobial activity of honey samples from Kosovo (antibacterial, antifungal, antiviral, and antimycobacterial properties) has been confirmed in *in vitro* and *in vivo* studies and was considered due to its acidity (low pH), osmotic effect, high sugar levels, and presence of bacteriostatic and bactericidal factors (hydrogen peroxide, antioxidants, lysozyme, polyphenols, phosphate, phenolic acid, and flavonoids) [11].

In particular, honey demonstrated high antimicrobial activity against different strains of *Staphylococcus* and *S*. *Typhimurium* ATCC 51812 [12].

It has been shown that undiluted and diluted honey at 75, 50, 30, and 10% is effective in inhibiting *S. aureus* and *S. epidermidis* [12]. Comparative research of antibacterial of honey in various dilutions to a number of commonly used antibiotics against nine pathogens from urine specimens found that honey samples tested are superior to all tested antibiotics [13]. Local honey obtained from Nigeria was tested *in vitro* against enteropathogen isolates; undiluted honey and different honey concentrations inhibit all the enteropathogens tested [14 ,15].

Bacteriostatic and bactericidal properties are identified in honey, against methicillin-resistant *Staphylococcus aureus* (MRSA), [16]. Honey activity against *Helicobacter pylori* (*H. pylori*) has been reported *in vitro* using agar diffusion assay [17].

Two enzymes, urease and xanthine oxidase, which are important virulence factors of *H. Pylori*, are effectively inhibited by honey phenolic components. These findings substantially confirm that regular consumption of honey (particularly polyphenol-rich foods) can help to prevent gastric ulcers due to *H. pylori* [18].

Different dilutions of honey samples collected from Ethiopia have both bacteriostatic and bactericidal activities against MRSA isolation from wound infection [19].

Strong antistaphylococcal honey activity has also been reported in MRSA clinical isolates (methicillin-resistant Staphylococcus aureus). Efficient inhibition growth was reported for MRSA isolates [20, 21]. Honey effectively eradicates *P. aeruginosa*-formed biofilm [22]. MRSA and *S*. *aureus* methicillin-sensitive (MSSA) measured honey was found to be susceptible with an inhibition zone of 36.2 ± 0.2 mm and 40.16 ± 0.152 mm, respectively [23].

Three honey samples were obtained from Baghdad tested for antibacterial activity. Three different concentrations were used 100, 70, and 50% against different organisms. The highest antibacterial activity of honey was reported on 100% concentration [24]. The activity of Danish honey was mostly considered due to hydrogen peroxide content [21].

The difference in honey's antibacterial activity depends on the floral origin of the honey [25]. Honey rich multiflora not only improves the nutritional quality and antimicrobial ability of several clinically significant microorganisms but also increases their nutritional potential [26]. Antimicrobial activity can be attributed to a variety of factors, in addition to the presence of hydrogen peroxide (H_2_O_2_), osmolarity, and acidity [27].

Water-dilute honeys (Sidr and Talh) had higher antimicrobial activity against bacterial strains than broad-spectrum antibacterial antibiotics (tetracycline and chloramphenicol), but they were less effective against fungal strains than antimicrobial antibiotics (Flucoral and mcosat) [28].

The aim of this study was to evaluate the antibacterial effect of global raw and commercial bee honey samples of different floral origins against 8 clinical isolates obtained from patients at private hospital from Sudan.

## 2. Materials and Methods

### 2.1. Bee Honey Samples

Thirty-two various global raw bee honey samples were obtained from different apiaries, as well as commercially sold honey samples from the local market, various brands, of different floral origins, between November and December 2018. The beekeepers determined the floral source of the honey based on the availability of flora for nectar foraging, the location of the apiary, and the honey's organoleptic qualities. Honey samples were stored in a sterile glass jar at room temperature. Samples were labeled according to the source, location, pH, date of collection, and floral origin as shown in Table 1.

### 2.2. Clinical Isolates

Eight clinical isolates were collected randomly from nose, throat, ear, wound, urine, stool, and conjunctival swabs, from both male and female patients at private hospital from Sudan (December, 2018), before antibiotic treatment. Sterile cotton swabs were used. The samples were immediately put in screw capped bottles containing transport medium. The collected isolates were used for conventional microbiological identification.

### 2.3. *In Vitro* Antibacterial Activity of Bee Honey

#### 2.3.1. Inoculum Preparation

Pure culture and standard inoculum size was maintained for antibacterial susceptibility. Clinical isolates were obtained from patients at private hospital (Sudan); were suspended in a sterile saline to match a 0.5 McFarland standard tube, which is accessible on the market; and provide a density of1.5 × 10^8^colony-forming units (CFU/ml).

#### 2.3.2. Disk Diffusion Susceptibility Test

The assay method was adapted from Allen et al. [29].

The 8 clinical isolates were identified by conventional microbiological methods and used for susceptibility test.

Mueller Hinton agar was utilized as the culture medium, reconstituted, sterilized (using autoclave) at 121°C for 15 minutes, left to set at 48°C, and inoculated with 0.1 ml of standardized 24 broth culture of bacterial suspensions that match the turbidity of the 0.5 McFarland standard tube (1.5 × 10^8^) (FU/ml). The guidelines for their use, as well as recommendations for how to use them, are provided by the National Committee for Clinical Laboratory Standards (NCCLS) (940 W. Valley Road, Suite 1400, Wayne, PA, 1987). The prepared Mueller Hinton medium was distributed aseptically in 20 ml volumes into sterile Petri dishes (95 mm internal diameter) and permitted to set. Sterile swabs were dipped in the fresh overnight microorganism suspension. The excess fluid was pressed against the wall tube then swabbed over the Mueller Hinton plates. Gently pressed over the top of the swabbed plate was the six-millimeter sterile filter paper disk impregnated with the honey sample (0.6 *μ*). The disk diffusion plate technique has been used to examine the antibacterial activity of bee honey.

The inoculated plates were incubated for 18-24 hours at 37°C. The diameter of the growth inhibition zone that resulted was measured in millimeter. In four replicates, each honey sample was examined against tested organism. The average diameter of the inhibition zone was measured.

#### 2.3.3. Epsilometer Test (Etest)

*(1) Predefined Gradient for MIC Determinations*. The Etest is a quantitative tool for assessing antimicrobial resistance of various bacterial strains. The method consists of an antibiotic gradient that is used to evaluate the minimum inhibitory concentration (MIC), in micrograms per milliliter, of various antibacterial agents against different microorganisms when measured on inoculated agar media.

A plastic strip that is thin, sterile, and nonporous, of gradient antibiotic , 5 mm wide and 60 mm long, is used in the Etest. The obtained result of MIC reading scale in grams per milliliter of the strip is printed on one side, with a two-letter code on the handle showing the exact antibiotic term, with the highest concentration at the top and lowest concentration at the bottom. The gradient spans 15 twofold dilutions of the traditional MIC form, covering a continuous concentration spectrum.

#### 2.3.4. Procedure for Etest

The following 6 Etests amoxicillin, ciprofloxacin, cefotaxime, chloramphenicol, gentamicin, and tetracycline were used against 8 clinical isolates: *S*. *aureus*, *E. coli*, *Klebsiella* spp., *Proteus vulgaris.*, *Salmonella* spp., *Shigella* spp., *Pseudomonas* spp., and methicillin-resistant *S*. *aureus* (MRSA).

Suspension of bacteria equivalent to a 0.5 McFarland tube was used to swab the surface of Mueller Hinton agar plate evenly, by sterile forceps. A strip of the Etest was removed and pressed on the inoculated plate; the whole strip is in total contact with the surface of the agar. For single MIC, two strips were used on a 90 mm agar plate. The inoculated medium was incubated at 37C° for 24 hours.

## 3. Results

Identification of the 8 clinical isolates obtained from patients at private hospital (Sudan) was carried out by the conventional microbiological methods at the Microbiology Laboratory. The following bacterial strains were identified as *Staphylococcus aureus*, methicillin-resistant *Staphylococcus aureus* (MRSA), *E*. *coli*, *K*. *pneumoniae*, *P*. *aeruginosa*, *P*. *vulgaris*, *S.* Typhi, and *S. sonnei*.

*In vitro* antibacterial effects against 8 clinical isolates, *S. aureus*, *E.coli*, *K. pneumoniae*, *P. aeruginosa*, *P. vulgaris*, *S.* Typhi, *S. sonnei*, and methicillin-resistant *S*. *aureus* (MRSA), were carried out by using 32 raw natural and commercial honeys. Both Gram-positive and Gram-negative bacteria were inhibited by 32 tested honey samples (Table 2).

Commercial honey samples exhibited antibacterial activity against all the 8 clinical isolates (Table 2).

In the present study, Gram-negative bacteria *P. aeruginosa* demonstrated the highest susceptibility towards different honeys tested. Sample D_1_ obtained from Damazine east of Sudan floral origin—sidr—was found to be the most active sample against *P. aeruginosa* (Table 2).

Antibacterial activity was observed in both natural and commercial honey samples against *Salmonella* Typhi (Table 2). Sample L_1_ obtained from Kashmir, floral origin sidr; sample L_2_ obtained from India; sample M from Iran commercial source; and sample X Jordanian allocation were revealed to be extremely effective against *Salmonella Typhi*, while samples C and D_1_ obtained from Sudan showed the least activity (Table 2).

*P*. *vulgaris*, *Klebsiella* spp., and *Shigella* spp. showed a consistent level of susceptibility to all honey tested. Samples D_1_, I_2_, U, and J showed the highest activity against *Proteus vulgaris* (Table 2). MRSA was subjected to antibacterial activity using the 32 honey samples and showed similar sensitivity to all honeys tested. Samples G, A,V, D_1_, and U exhibited the highest activity (Table 2). The tested antibiotics against different organisms showed low MIC (Table 3). On the other hand, most of the clinical isolates were consistently found susceptible to all honeys tested.

## 4. Discussion

Controlling the rise in antibiotic resistance is one of the most pressing issues facing modern health care. Resistance to frequently used community antimicrobials is rising, according to international resistance surveillance studies. Honey has been valued for its medicinal properties since ancient times. The topical application of honey, as wound dressing, has got a lot of attention in recent years. Honey has been applied successfully to treat infections that have stopped responding to traditional antiseptic and antibiotic care. Honey has different types and levels of antibacterial activity depending on the floral source. The obtained previous favorable results previous of antibacterial activity of honey against standard organisms, it is encouraged to explore the activity of honey against clinical isolates [30].

*In vitro* antibacterial activity of honey against 8 clinical isolates showed different susceptibility patterns on both Gram-positive and Gram-negative bacteria. *S. aureus* was found to be the most sensitive to different honey samples. The present findings confirmed that the 12 commercial honey samples with different brands exhibited antibacterial activity against the 8 clinical isolates (Table 2). This proved that commercial honey has the same activity as the raw natural unprocessed honey. Previous studies documented that Australian Jelly bush honey is a commercial honey that has been marked as bioactive honey [5].

The actual impact of bee honey's geographical origin on its antibacterial activity was more pronounced in the case of *S. aureus* as sample I_2_ obtained from Saudi Arabia floral origin. Sunflower was found to be more effective against different tested organisms (Table 2). The content of phenolic acid in honey is affected by geographical location and the source of nectar plants [3]. These data imply that the connection between antibacterial activity, floral source, and environmental variables varies by geographical area [3].

Our results revealed that both natural and commercial honeys showed identical sensitivity towards the most tested microorganisms. Antibacterial activity against *S.* Typhi, *P*. *vulgaris*, *Klebsiella*, and *Shigella* exhibited similar sensitivity to all honeys tested. These results seems in agreement with the previous findings [5]. *S*. Typhi was noted to be sensitive to the most of honey tested [12, 30]. Bacterial adhesion to epithelial cells in the mucosa is thought to be the first stage in the enhancement of a bacterial infection of the gastrointestinal tract. Since disrupting pathogenic microorganisms' attachment to the intestinal epithelium is a possible technique for controlling disease, honey is useful in the treatment of enteric pathogens, as well as inhibiting their growth and reducing their attachment to the epithelial cells of the gastrointestinal tract [28].

MRSA was subjected to antibacterial activity using the 32 honey samples and showed similar sensitivity to all honeys tested. Sample B, G, V, and P exhibited the highest activity against MRSA. These are in line with the previous findings which confirmed the effectiveness of different honey samples against MRSA [6, 17, 20, 21]. MRSA has been a frequent cause of hospital-acquired infection and a major cause of serious infection, as well as had a pattern of resistance, not only to methicillin but also to aminoglycoside and cephalosporin [29].

The geographical and floral origin of honey samples was more pronounced on the result obtained with sample D_2_ Damazine east of Sudan, floral origin; sidr against *Pseudomonas* demonstrated the highest activity. The influnence of geographical source of bee hone on its antibacterial activity wasmore pronounced against Pseudomonas with sample D2-east of Sudan floral orgin sidr. Honey effectively eradicates *P. aeruginosa*-formed biofilm [22]. *Pseudomonas* spp. were well known, as a multi-drug-resistant organism. It was previously thought to be the most common cause of hospital-acquired infection. This wide range of honey sample inhibitory effects suggests differences in antimicrobial constituent (s) of honey. Similar previous findings referred the antimicrobial property of honey to different constituents [11]. Significant antibacterial activity of different floral honeys was proved against a wide range of Gram-positive and Gram-negative bacterial standard organisms [30]. The promising effects of our previous investigation on honey's antibacterial activity against standard organisms encourged to examine honey's antibacterial activity against clinical isolates.

The clinical isolates were tested for their *in vitro* sensitivity test against the 8 antibiotics. Bacterial strains showed low-sensitivity MIC towards antibiotics tested, as compared with honey antibacterial activity (Table 3).

The current study highlights the susceptibility of *Pseudomonas* spp. to the most honey samples (raw and commercial). *Pseudomonas* spp. among the Gram-negative bacteria revealed resistance to the majority of antibiotics tested [30]. *P*. *aeruginosa* was a common infection seen in hospitals. Honey was reported to be more effective than antibiotics at controlling bacterial growth. In the present research, raw and commercial honey has been found to be a potent antimicrobial natural product against a variety of microorganisms, including multiresistant strains. These observations were concomitant with the findings of the previous studies [22, 23, 28]. Honey has been used effectively to treat chronic infections that have failed to react to standard antiseptics and antibiotics. These criteria show promise in the treatment of bacterial infections, especially in the clinical treatment of ulcers, bed sores, burns, fractures, and surgical wounds. Honey's antimicrobial properties may be especially beneficial against bacteria that have developed antibiotic resistance. Broad-spectrum antimicrobial activity of honey can be attributed to a variety of factors, in addition to the presence of hydrogen peroxide (H_2_O_2_), osmolarity, and acidity [27]. Our results revealed that the antibacterial activity of different honey samples could explain the floral diversity according to the geographic location. A previous study confirmed that difference in honey's antibacterial activity depends on the floral origin of the honey [25]. The current study highlights that tested antibiotics showed selective inhibition of the clinical isolates, which was not the case with the honey samples. Honey superiority over antibiotics over a wide variety of microbes was reported. These results are in accordance with a previous comparative study of honey to a number of commonly used antibiotics against 9 pathogens isolated from urine specimens and showed that tested honey samples is superior to all antibiotics [13].

Honey has also been reported to be more effective as an antibacterial agent against several *Pseudomonas* and *Staphylococcus* strains than antibiotic gentamicin [28]. To demonstrate the synergistic action of honey and antibiotics, honey can be given orally with antibiotics [10]. The inhibitory impact of various floral honey samples against resistant microbes that taint wounds and hinder wound healing would pave the way for honey to be reintroduced into modern medicine. The limitation of honey's medical usefulness *in vivo* is being studied for future medical practice. To produce the active component as a pharmaceutical product, more research on the active compounds of efficient antibacterial action of natural bee honey is required. Hence, there is a need to find natural alternative treatment to counter the multi-drug-resistant organisms. A traditional therapy therefore appears to have enormous potential in solving new clinical problems. To assess the potency of raw natural and commercial honey against isolated clinical isolates, further investigations should be conducted against antibiotic-resistant microorganisms.

## 5. Conclusion

Both natural and commercial honey samples exerted inhibitory effects on the various Gram-positive and Gram-negative bacteria understudy. Commercial honey has the same activity as the raw natural unprocessed honey. Honey was found to be effective in inhibiting *P*. *aeruginosa* and MRSA. Thus, honey is highly recommended as wound dressing to manage wound healing due to its antibacterial activity against a wide range of microorganisms. Geographical and botanical sources were more pronounced on honey antibacterial properties. Antibacterial activity in honey is influenced by a variety of factors that are not entirely dependent on the floral source. Honey could be an alternative treatment approach in chronic wounds and burns of different natures in inhibiting different types of microorganisms not responding to conventional antibiotics without side effects. Further research efforts would be beneficial in attempt to control resistant organisms not responding to antibiotic treatment. Using honey in a medical setting may reduces financial cost and hospital stay.

## Figures and Tables

**Table 1:** Thirty-two different global honey samples collected from different countries.

Code	Locality	Source	Floral origin pH
A	Sudan	East Singa	Neem 3.4
B	Sudan	East Singa	Sidr 4.6
C	Sudan	East Singa	Sidr 4.9
D_1_	Sudan	Damazine east	Sidr 4.7
D_2_	Sudan	West apiary	Acacia 4.3
E	Sudan	West Darfur apiary	Sidr 4.5
F	Sudan	West Darfur apiary	Mountain 4.6
G	Sudan	South apiary	Acacia 4.2
H	Sudan	Darfur apiary	Acacia 3.5
I_1_	Saudi Arabia	College of Agriculture apiary	Sun flower 3.5
I_2_	Saudi Arabia	College of Agriculture apiary	Sun flower 3.5
J	Kashmir	Commercial	Sun flower 3.5
K	Yemen	Apiary	Sidr 3.5
L_1_	Kashmir	Apiary	Sidr 3.5
L_2_	India	Apiary	Unknown 3.5
M	Iran	Commercial	Sidr 4.2
N	Turkey	Commercial	Orange 3.5
O	Saudi Arabia	Al-Shifa commercial	Flowers 4.2
P	Australia	Commercial	Flowers 4.1
Q	Argentine	Commercial	Orange 4.3
R	Saudi Arabia	Al-Shifa commercial	Orange 4.0
S	United stated	Goody commercial	Flowers 3.8
T_1_	Germany	Langnese commercial	Flowers 3.7
T_2_	Germany	Langnese commercial	Flowers 3.7
U	Saudi Arabia	Al-Shifa commercial	Acacia 4.2
V	Palestinian	Apiary	Citrus 4.2
W_1_	Egypt	Apiary	Alfa 4.6
W_2_	Egypt	Apiary	Alfa 4.5
X	Jordan	Apiary	Citrus 4.5
Y_1_	Yemen	Apiary	Sidr 4.4
Y_2_	Yemen	Commercial	Sidr 4.5
Z	Saudi Arabia	Tabuk apiary	Sun flower 4.3

**Table 2:** Antibacterial activity of 32 bee honeys against 8 clinical isolates.

Honey samples	Bacterial strain							
	*S. aureus*	*E. coli*	*K. pneumoniae*	*P. aeruginosa*	*Proteus*	*S.* Typhi	*S. sonnei*	MRSA
	Inhibition zone in mm ± (SD)							

A	25 ± (0.5)	22 ± (0.5)	22 ± (0.5)	25 ± (0.5)	23 ± (0.5)	21 ± (0.5)	22 ± (0.5)	24 ± (0.5)
B	25 ± (0.7)	20 ± (0.5)	21 ± (0.6)	24 ± (0.6)	22 ± (0.5)	22 ± (0.5)	23 ± (0.5)	25 ± (0.6)
C	24 ± (0.5)	24 ± (0.5)	21 ± (0.5)	31 ± (0.6)	24 ± (0.5)	19 ± (0.5)	24 ± (0.5)	23 ± (0.6)
D_1_	22 ± (0.5)	19 ± (0.5)	20 ± (0.5)	(0.5)38±	25 ± (0.6)	20 ± (0.5)	22 ± (0.5)	22 ± (0.5)
D_2_	24 ± (0.5)	20 ± (0.5)	21 ± (0.5)	27 ± (0.4)	24 ± (0.4)	25 ± (0.5)	24 ± (0.4)	21 ± (0.5)
E	(0.5)24±	19 ± (0.5)	20 ± (0.5)	28 ± (0.6)	23 ± (0.5)	22 ± (0.4)	23 ± (0.6)	22 ± (0.4)
F	25 ± (0.6)	20 ± (0.5)	21 ± (0.5)	26 ± (0.5)	22 ± (0.4)	22 ± (0.5)	22 ± (0.4)	24 ± (0.5)
G	23 ± (0.5)	25 ± (0.4)	22 ± (0.5)	25 ± (0.4)	23 ± (0.5)	23 ± (0.4)	21 ± (0.4)	25 ± (0.5)
H	24 ± (0.4)	22 ± (0.5)	21 ± (0.4)	21 ± (0.4)	23 ± (0.5)	21 ± (0.5)	20 ± (0.5)	23 ± (0.5)
I_1_	26 ± (0.5)	23 ± (0.6)	22 ± (0.4)	23 ± (0.6)	24 ± (0.4)	22 ± (0.4)	22 ± (0.4)	24 ± (0.6)
I_2_	27 ± (0.5)	22 ± (0.5)	23 ± (0.6)	23 ± (0.5)	25 ± (0.5)	23 ± (0.6)	23 ± (0.5)	21 ± (0.5)
J	23 ± (0.6)	21 ± (0.5)	22 ± (0.5)	25 ± (0.6)	26 ± (0.5)	24 ± (0.5)	25 ± (0.5)	22 ± (0.5)
K	24 ± (0.4)	18 ± (0.5)	23 ± (0.6)	24 ± (0.4)	24 ± (0.6)	25 ± (0.6)	22 ± (0.4)	23 ± (0.5)
L_1_	22 ± (0.4)	17 ± (0.4)	23 ± (0.5)	24 ± (0.4)	24 ± (0.5)	23 ± (0.4)	21 ± (0.5)	24 ± (0.4)
L_2_	22 ± (0.6)	22 ± (0.5)	22 ± (0.5)	22 ± (0.4)	25 ± (0.5)	24 ± (0.4)	23 ± (0.5)	22 ± (0.4)
M	23 ± (0.5)	22 ± (0.5)	23 ± (0.5)	20 ± (0.4)	23 ± (0.5)	22 ± (0.4)	24 ± (0.5)	21 ± (0.4)
N	22 ± (0.5)	23 ± (0.5)	22 ± (0.5)	24 ± (0.4)	24 ± (0.5)	23 ± (0.5)	23 ± (0.5)	23 ± (0.5)
O	21 ± (0.4)	20 ± (0.5)	23 ± (0.5)	23 ± (0.4)	24 ± (0.5)	21 ± (0.4)	22 ± (0.5)	25 ± (0.4)
P	23 ± (0.5)	21 ± (0.5)	23 ± (0.5)	23 ± (0.5)	23 ± (0.4)	22 ± (0.4)	21 ± (0.4)	24 ± (0.4)
Q	24 ± (0.5)	20 ± (0.5)	21 ± (0.6)	23 ± (0.5)	25 ± (0.5)	23 ± (0.6)	22 ± (0.5)	23 ± (0.5)
R	23 ± (0.6)	21 ± (0.5)	22 ± (0.4)	22 ± (0.5)	22 ± (0.5)	23 ± (0.4)	23 ± (0.4)	22 ± (0.5)
S	24 ± (0.5)	22 ± (0.5)	24 ± (0.4)	23 ± (0.5)	23 ± (0.6)	22 ± (0.5)	24 ± (0.6)	21 ± (0.4)
T_1_	25 ± (0.4)	20 ± (0.5)	22 ± (0.6)	22 ± (0.5)	24 ± (0.5)	24 ± (0.5)	23 ± (0.5)	24 ± (0.5)
T_2_	24 ± (0.6)	21 ± (0.5)	21 ± (0.4)	25 ± (0.5)	25 ± (0.4)	23 ± (0.5)	22 ± (0.5)	24 ± (0.6)
U	25 ± (0.5)	22 ± (0.6)	21 ± (0.5)	24 ± (0.5)	24 ± (0.5)	22 ± (0.5)	23 ± (0.4)	25 ± (0.5)
V	23 ± (0.5)	21 ± (0.4)	22 ± (0.5)	25 ± (0.4)	22 ± (0.5)	24 ± (0.6)	22 ± (0.5)	23 ± (0.6)
W_1_	22 ± (0.5)	22 ± (0.5)	20 ± (0.5)	24 ± (0.5)	21 ± (0.5)	22 ± (0.4)	21 ± (0.5)	22 ± (0.5)
W_2_	21 ± (0.5)	20 ± (0.6)	21 ± (0.5)	23 ± (0.6)	23 ± (0.5)	21 ± (0.5)	22 ± (0.5)	21 ± (0.4)
X	24 ± (0.6)	22 ± (0.4)	22 ± (0.5)	22 ± (0.5)	24 ± (0.4)	22 ± (0.5)	23 ± (0.5)	22 ± (0.6)
Y_1_	23 ± (0.5)	21 ± (0.5)	21 ± (0.5)	23 ± (0.5)	22 ± (0.5)	22 ± (0.6)	24 ± (0.5)	23 ± (0.5)
Y_2_	22 ± (0.4)	20 ± (0.5)	21 ± (0.4)	22 ± (0.6)	21 ± (0.5)	21 ± (0.5)	23 ± (0.5)	21 ± (0.4)
Z	23 ± (0.5)	22 ± (0.4)	21 ± (0.4)	22 ± (0.5)	21 ± (0.5)	23 ± (0.5)	22 ± (0.4)	21 ± (0.5)

**Table 3:** Minimum inhibitory concentration of 6 antibiotics against 8 clinical isolates (Etest).

Antibiotics	Bacterial strains							
	*E. coli*	*Klebsiella*	*Proteus vulgaris*	*Salmonella*	*Shigella*	*Pseudomonas*	MSSA	MRSA
	MIC (mg/l)							

Amoxicillin	3	3	0.125	1.5	0.6	3	4	0.125
Ciprofloxacin	2	0.125	0.5	2.5	0.18	0.125	3	0.50
Cefotaxime	0.5	0.6	0.125	0.6	0.125	0.125	3	1.0
Chloramphenicol	1.5	3	0.50	3	2	1.5	0.125	3
Gentamicin	3	1.5	0.50	0.125	1.5	0.125	3	3
Tetracycline	4	1.5	0.064	0.125	0.50	0.50	0.125	1.0

## Data Availability

The datasets used and analyzed in the current study are included in the article.
